# Development of oral cancer tissue-mimicking phantom based on polyvinyl chloride plastisol and graphite for terahertz frequencies

**DOI:** 10.1117/1.JBO.25.12.123002

**Published:** 2020-11-17

**Authors:** Tianmiao Zhang, Ravshanjon Nazarov, Alexey P. Popov, Petr S. Demchenko, Alexander V. Bykov, Roman O. Grigorev, Anna V. Kuzikova, Victoria Y. Soboleva, Dmitry V. Zykov, Igor V. Meglinski, Mikhail K. Khodzitskiy

**Affiliations:** a ITMO University, School of Photonics, Terahertz Biomedicine Laboratory, Saint Petersburg, Russia; b Tydex LLC, Saint Petersburg, Russia; c University of Oulu, Faculty of Information Technology and Electrical Engineering, Optoelectronics and Measurement Techniques Laboratory, Oulu, Finland; d Aston University, Aston Institute of Materials Research, School of Engineering and Applied Science, Birmingham, United Kingdom; e Aston University, School of Life and Health Sciences, Birmingham, United Kingdom

**Keywords:** phantom, effective medium theory, optical property, terahertz, oral tissue, cancer

## Abstract

**Significance:** A new concept of a biotissue phantom for terahertz (THz) biomedical applications is needed for reliable and long-term usage.

**Aim:** We aimed to develop a new type of biotissue phantom without water content and with controllable THz optical properties by applying graphite powders into a polyvinyl chloride plastisol (PVCP) matrix and to give a numerical description to the THz optical properties of the phantoms using the Bruggeman model (BM) of the effective medium theory (EMT).

**Approach:** The THz optical properties of graphite and the PVCP matrix were measured using THz time-domain spectroscopy, which works in the frequency range from 0.1 to 1 THz. Two phantoms with 10% and 12.5% graphite were fabricated to evaluate the feasibility of describing phantoms using the EMT. The EMT then was used to determine the concentration of graphite required to mimic the THz optical properties of human cancerous and healthy oral tissue.

**Results:** The phantom with 16.7% of graphite has the similar THz optical properties as human cancerous oral tissue in the frequency range of 0.2 to 0.7 THz. The THz optical properties of the phantom with 21.9% of graphite are close to those of human healthy oral tissue in the bandwidth from 0.6 to 0.8 THz. Both the refractive index and absorption coefficient of the samples increase with an increase of graphite concentration. The BM of the EMT was used as the numerical model to describe the THz optical properties of the phantoms. The relative error of the BM for the refractive index estimation and the absorption coefficient is up to 4% and 8%, respectively.

**Conclusions:** A water-free biotissue phantom that mimics the THz optical properties of human cancerous oral tissue was developed. With 21.9% of graphite, the phantom also mimics human healthy oral tissue in a narrow frequency range. The BM proved to be a suitable numerical model of the phantom.

## 1 Introduction

Terahertz (THz) technology has been applied in many scientific areas, such as explosive detection, quality control, and medical diagnostics.^1^^–^^9^ Compared with other medical diagnosis methods, THz technology has many advantages. THz radiation does not cause ionization in biotissues because it has very low photon energy.^2^^–^^6^ Since THz radiation is very sensitive to water and the hydration state of the object, which leads to good contrast between the normal and diseased tissues, THz has great promise in medical applications, especially for cancer diagnosis and tumor resection.^4^^,^^5^^,^^8^^–^^10^

However, the inhomogeneity of biological tissues still causes difficulties for the classification of healthy tissues and tumors.^8^^,^^9^ There is a failure in about 15% to 20% of all cases to remove the cancer with enough margin.^11^ Thus, a clear understanding of how each tissue component and its concentration may affect the optical properties of a biological tissue is necessary for the development of cancer diagnosis. It is impossible to control the component concentrations in real biological tissues and organs. Furthermore, the properties of freshly excised tissues change over time due to the alteration of their natural environment. Therefore, tissue model phantoms that have similar optical properties of real biological objects are needed. Moreover, proper phantoms can ensure data accuracy and consistency over time, among multiple instruments, and across different device manufacturers. This is a key step toward standardization and quality assurance.

Several types of THz phantoms have been developed in recent works. Phantoms that were composed of water, oil, surfactant TX151 (a polysaccharide material), agar, nano-diamonds, and nano-onions have been designed to represent the optical properties of breast tissue in the THz frequency range,^12^^–^^14^ as well as phantoms that were composed of water, lipid, and gelatin^15^ and phantoms with TX151 gel and napthol dye.^16^ Additionally, a phantom consisting of porcine-derived gelatin and water was tested.^17^^,^^18^ However, these phantoms are not stable since water evaporation affects their optical properties. A water-free phantom needs to be developed for stable and long-term usage. In the previous research, a polyvinyl chloride plastisol (PVCP) based phantom was fabricated to mimic the refractive index of human hand skin,^18^ yet phantoms for other tissues need to be developed.

The most important criterion for disease detection in THz frequencies is to have clearly distinguishable gaps between THz optical properties (refractive index, absorption coefficient, and permittivity) of healthy and diseased tissues. For oral cancer, the gap of the refractive index is in the frequency range of 0.2 to 0.8 THz, and the gap of the absorption coefficient is in the frequency range of 0.4 to 1 THz.^10^ Thus, the optimal bandwidth for oral cancer diagnosis is 0.4 to 0.8 THz, and this is the target frequency range for the phantom design.

This study aims to advance existing findings of a water-free phantom for oral tissue with different concentrations of graphite. The accurate optical properties of each component were measured for graphite concentration estimation. The Bruggeman model (BM) from the effective medium theory (EMT) was used to predict and determine the graphite concentration for mimicking the optical properties of human cancerous oral tissue as well as healthy oral tissue in the frequency range from 0.4 to 0.8 THz. The reliability of the BM for the estimation of THz optical property of phantoms with different graphite concentrations was also investigated.

## 2 Experiments and Methodology

### 2.1 Sample Preparation

Traditionally, a biotissue phantom consists of transparent or low scattering matrix/base material and absorbers at different concentrations mimicking the refractive and absorption properties of real tissues. PVCP is one of the popular choices for biotissue phantoms as it is a matrix material in the visible and infrared frequencies.^19^^,^^20^ PVCP in general conditions is liquid and can be solidified by heating. Due to its good flexibility and long-term physical stability, it is worth investigating its application at THz wavelengths as a phantom matrix. Inspired by a prior article,^21^ graphite powder from a 9B graphite stick was chosen as the absorber of the phantom since it has a high absorption coefficient in THz frequency range, which may compensate for the weak absorbance of PVCP.

The general fabrication steps begin with mixing PVCP and graphite powder together. After sonicating the mixture for 15 min, graphite power was evenly spread in the PVCP liquid, and the mixture became emulsion. Then the emulsion was poured into a rectangular aluminum mold and put into an oven (180°C) to be heated for 30 min. Finally, after cooling, the emulsion became solid and the phantom was fabricated. The phantom was stored between glass slides to prevent PVC penetration into the plastic petri dish.^20^

The thickness of the phantom was measured by a confocal microscope. The volume concentration of graphite powder could not be directly measured. The following formula was used to calculate the concentration indirectly using the mass:

$$\delta_{g}=\frac{V_{g}}{V_{ph}}=\frac{V_{g}}{V_{m}+V_{g}}=\frac{\frac{m_{g}}{\rho_{g}}}{V_{m}+\frac{m_{g}}{\rho_{g}}},$$ (1)

where $V_{g}$, $V_{m}$, and $V_{ph}$ are the volume of graphite, matrix, and phantom, respectively; $m_{g}$ is the mass of graphite powder; $\rho_{g}$ is the density of graphite; and $\delta_{g}$ is the volume concentration of graphite, among them $V_{m}$, $m_{g}$, and $\rho_{g}$ are known.

### 2.2 Experiment Setup

**Fig. 1:**
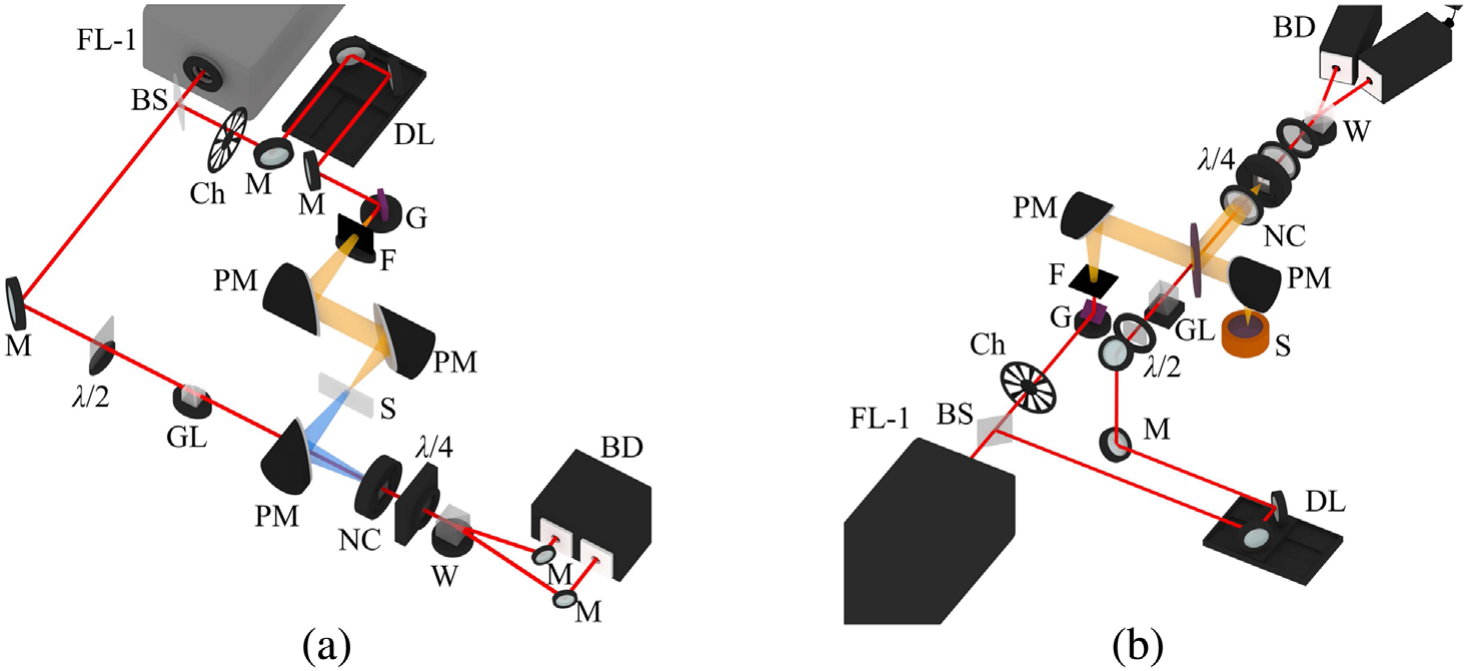
The experimental setup of the THz-TDS system in (a) transmission mode and (b) reflection mode. FL-1–femtosecond laser, BS–beam splitter, Ch–chopper, M–mirror, DL–optical delay line, G–InAs antenna, F—infrared filter, PM–parabolic mirror, S–sample, λ/2–half-wave plate, GL—Glan prism, L—lens, NC—CdTe crystal, λ/4–quarter-wave plate, and W–Wollaston prism, BD—balance detector.

**Fig. 2:**
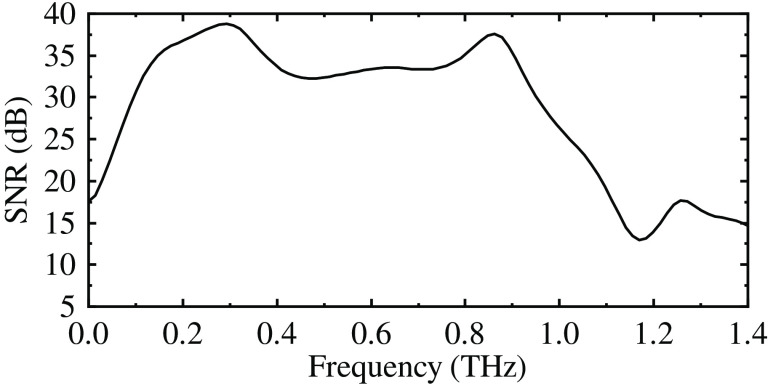
The SNR of the THz-TDS setup.

To get optical properties of the phantoms, THz time-domain spectroscopy (TDS) was used. For the measurement of the phantom matrix and water-free phantom, THz-TDS in transmission mode was used. For the measurement of graphite, since THz radiation can not penetrate the graphite stick, THz-TDS in reflection mode was used. The outline of the THz-TDS setup is shown in Fig. 1.^22^ A femtosecond laser beam was generated in a series of pulses (1040 nm, 200 fs, 70 MHz, 15 nJ, Solar Laser Systems, Belarus). The beam was then divided into two beams by a beam splitter. One beam passed through a time delay line and then hit the indium arsenide (InAs) semiconductor antenna to generate THz radiation. The THz radiation then transmitted through or reflected on the sample and reached the cadmium telluride (CdTe) semiconductor crystal. Meanwhile, another beam, known as a probe beam, having passed through a half-wave plate and a Glan prism, finally met with the THz beam that transmitted through the sample on the CdTe surface to have its polarization changed. The polarization change of each probe beam was associated with a certain time. By detecting orthogonally polarized components of the probe beam using a balanced photodetector, the waveform of the THz beam was finally recorded. The output power of the THz-TDS setup was up to 300 nW. The highest frequency resolution was 2 GHz. The frequency range (in air) was 0.2 to 1.2 THz (Fig. 2). In this paper, the frequency resolution of the measurements was set as 20 GHz.

### 2.3 Data Acquisition and Parameter Extraction

To perform the measurements using the THz-TDS setup in transmission mode, first, a reference signal was obtained while no sample was placed in the TDS setup. Then samples were placed on the sample mount and were measured at ambient temperature (20°C) one by one. For each sample, five measurements were performed continuously. Then the averaged waveforms were used to calculate the optical properties of the samples to diminish the random and optical delay line positioning errors. The waveforms were first filtered using the Gaussian window to acquired correct phases.^23^ After the filtration, the Fourier transform was done to extract information such as amplitude and phase of the signals. The refractive index and absorption coefficient of the sample were calculated using the following formulas for the transmission measurements:^24^^–^^26^

α(v)=−2d ln[|E^sample(v)|T(v)|E^reference(v)|], (2)

$$n(v)=1+c[\phi_{\text{sample}}(v)-\phi_{\text{reference}}]/2\pi vd,$$ (3)

$$T(v)=1-R(v)=1-{\left[n(v)-1\right]}^{2}/{\left[n(v)+1\right]}^{2},$$ (4)

where α is the absorption coefficient of the sample; n is the refractive index of the sample; $\hat{E}$ and ϕ are the amplitude and the phase of the signal, respectively; v is a frequency; d is the thickness of the sample; R is the Fresnel loss (reflectance) at the air–sample interface; and c is the speed of light.

**Fig. 3:**
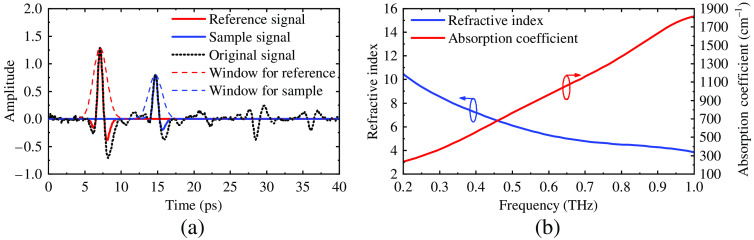
(a) The measured refractive index and absorption coefficient of graphite and (b) the acquired signal using the THz-TDS setup in reflection mode The red and blue dash lines indicate the Gaussian windows that were used to extract the reference signal and the sample signal from the original waveform (the black dash-dot line)

For the measurement of graphite using THz-TDS in reflection mode, the graphite stick was mounted and covered by a dielectric lossless window made of silicon. The THz beam reached the surface of the window at normal incidence; then the THz beam was reflected at the air–window interface and window–sample interface. Thus, in one measurement, two pulses were recorded, as shown in Fig. 3(b). The reference and sample signals were extracted using Gaussian windows. Similar to the measurement of the phantoms, five measurements for each tissue sample were carried out to calculate the average amplitude of the THz waveform. For the extraction of the optical properties obtained from the reflection mode, the following formulas were used:^27^

$$n_{s}(v)=\frac{n_{w}(1-A^{2})}{1+A^{2}+2A\;\cos\;\phi },$$ (5)

$$\alpha_{s}(v)=\frac{2n_{w}A\;\sin\;\phi }{1+A^{2}+2A\;\cos\;\phi }\cdot \frac{4\pi v}{c},$$ (6)

A exp(iϕ)=1−nw24nw·exp[2jnw2πvdwc]·E^sample(v)E^reference(v), (7)

where $n_{s}$ and $n_{w}$ are the refractive index of the sample and dielectric window; respectively; $\alpha_{s}$ is the absorption coefficient of the sample; v is a frequency; E^reference and E^sample are the complex amplitudes of the reference and sample signals, respectively; ϕ is the phase of Eq. (7); $d_{w}$ is the thickness of the window; and c is the speed of light.

### 2.4 Analysis Methodology—Effective Medium Theory

The optical properties of heterogenous mixtures can be estimated effectively using the EMT.^28^ In previous studies, one of the EMT models––BM proved to be suitable for two-component mixtures compared with the other EMT models.^29^^,^^30^ The BM in general has three approaches depending on the shape of the components. Since which approach is suitable for the estimation is unknown, phantoms with the graphite concentrations of 10% and 12.5% were fabricated to evaluate their feasibility. The phantoms were first measured using THz-TDS in transmission mode; then three types of BMs were used to estimate the THz optical properties of each phantom using those of the PVCP matrix and graphite. By comparing estimated and measured values, the BM that has the best fit was chosen to determine the graphite concentrations for the phantoms of healthy and cancerous oral tissues. Results are demonstrated in Sec. 3 and Fig. 5. Once the approach of the BM was determined, the estimated concentration of graphite was calculated reversely for the fabrication of the oral tissue phantoms. The three approaches of the BM are as shown below:^28^

BM for spherical particles:

ε^R−ε^h3ε^R=fpε^p−ε^hε^p+2ε^R. (8)

BM for discoid particles:

1−fp=(ε^p−ε^Rε^p−ε^h)(2ε^p+ε^h2ε^p+ε^R). (9)

BM for rod-shaped particles:

1−fp=(ε^p−ε^Rε^p−ε^h)(5ε^h+ε^p5^εR+ε^p)25, (10)

where $f_{p}$ is the volumetric concentration of particles, ${\hat{\varepsilon }}_{p}$ is the complex permittivity of particles, ${\hat{\varepsilon }}_{h}$ is the complex permittivity of the host material, and ${\hat{\varepsilon }}_{R}$ is the effective complex permittivity of the composite medium.

## 3 Results and Discussions

**Fig. 4:**
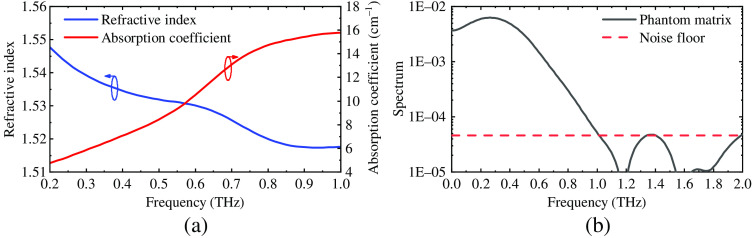
(a) The measured refractive index, absorption coefficient and (b) spectrum of phantom matrix (PVCP) with the thickness of 5 mm. The noise floor shows that the reliable frequency range of the measurements is 0.2 to 0.8 THz.

The measured frequency-dependent refractive indices and absorption coefficients of graphite and the phantom matrix are shown in Figs. 3 and 4. According to the literature,^27^ the lowest and highest frequencies of reliable measurements are determined by the signal-to-noise ratio (SNR), and the highest frequency of reliable measurements is determined by the maximum absorption coefficient. The lowest frequency is the point where the “flat SNR” starts, i.e., according to Fig. 2, 0.2 THz.

**Fig. 5:**
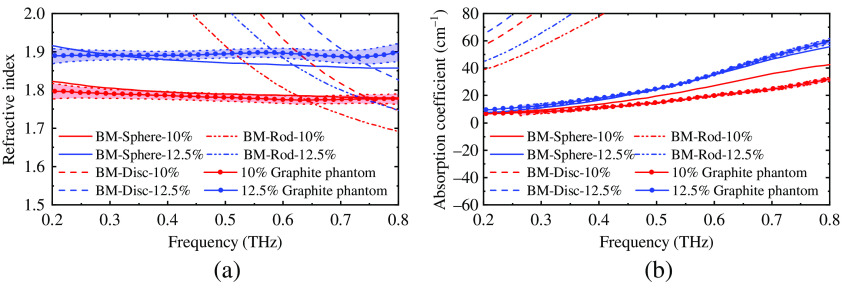
The (a) estimated and (b) measured optical properties of phantoms with 10% and 12.5% graphite. The estimation was calculated using BMs for spherical, discoid, and rod-shaped particles. It is clear that the BM for spherical particles matches the experimental data precisely.

**Fig. 6:**
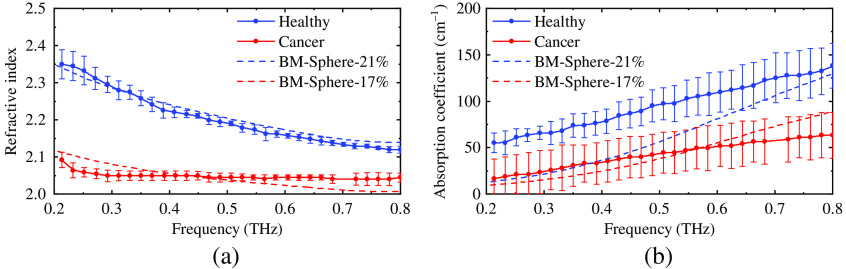
The comparison of the estimated (a) refractive index and (b) absorption coefficient of phantoms with 21% and 17% graphite (solid lines) and the literature values of healthy and cancerous oral tissue^10^ (dash lines).

**Fig. 7:**
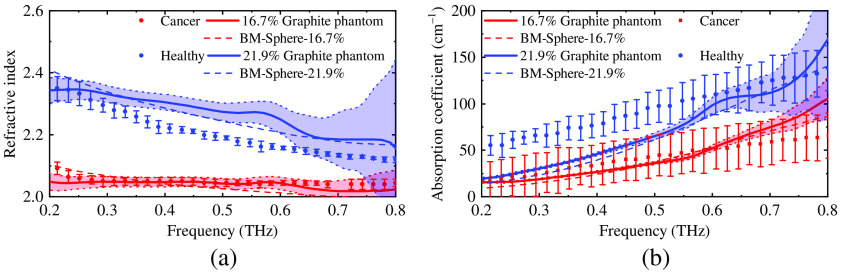
The comparison of the estimated (dash-dot lines) and measured (solid lines) optical properties [(a) refractive index and (b) absorption coefficient] of phantoms and the literature values of healthy and cancerous oral tissue^10^ (dot lines).

As mentioned in Sec. 2.4, evaluations need to be made to choose the most suitable approach of the BM. The estimated and measured properties of the phantom with 10% and 12.5% graphite are compared in Fig. 5. The BM for spherical particles has the best fit to the measured properties of the phantoms. Thus, it was used to determine the graphite concentrations for the phantoms of healthy and cancerous oral tissues. Figure 6 shows that phantom with 17% and 21% graphite has the most similar optical properties of cancerous and healthy oral tissue, respectively, according to the estimation. Due to the measurement error of the graphite mass, the phantoms with 16.7% and 21.9% graphite were fabricated in the end. Then, the optical properties of the phantoms were extracted from experiments; in Fig. 7 they are compared with literature values for healthy and cancerous oral tissues^10^ and the estimated values obtained using the BM.

**Fig. 8:**
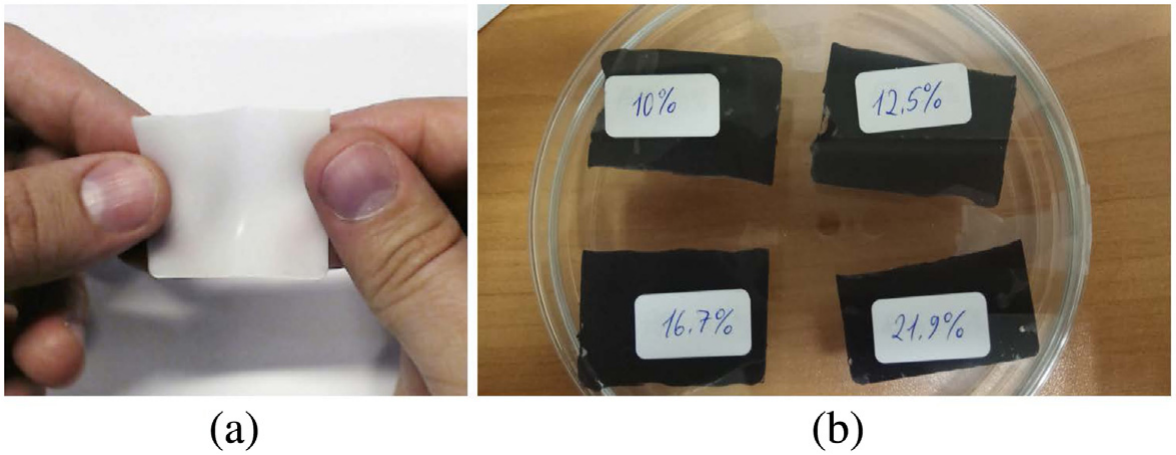
The phantom matrix (a) was made of PVCP. The fabricated PVCP-graphite-based phantoms (b) were made of PVCP and graphite with volume concentration of 10%, 12.5%, 16.7%, and 21.9%.

Figure 8(a) shows the good flexibility of the PVCP matrix. Figure 8(b) shows the fabricated phantoms placed in a plastic petri dish. Table 1 provides the compositions and the thicknesses of all fabricated phantom.

**Table 1:** Composition of two-component phantoms.

Sample	%VoL Graphite	%VoL PVCP	Thickness (mm)
1	10.0	90.0	0.55±0.005
2	12.5	87.5	0.7±0.005
3	16.7	83.3	0.65±0.005
4	21.9	78.1	0.57±0.005

### 3.1 Graphite

Experiment results show that graphite may significantly increase the value of a phantom’s optical properties. Figure 7 shows the THz optical properties of the phantoms and the tissues. A The phantom with 16.7% of graphite has notably similar optical properties of cancerous oral tissue in the frequency range of 0.2 to 0.7 THz. The refractive index of the phantom with 21.9% of graphite is higher that of healthy oral tissue for about 0.1 in the whole bandwidth, and the absorption coefficient of the phantom has similar values as that of healthy tissue in a narrow frequency range of 0.6 to 0.8 THz, which still lies inside the target bandwidth.

It is hard to achieve a similar dispersion of the optical properties of water, the most common element in human tissues, simply using graphite and PVCP. The phantoms with graphite have much lower absorption coefficients in the lower frequency range (0.2 to 0.7 THz) when comparing with many other human tissues.^31^ By far, only oral tissues can be mimicked by our phantoms with graphite.

### 3.2 Bruggeman Model

**Fig. 9:**
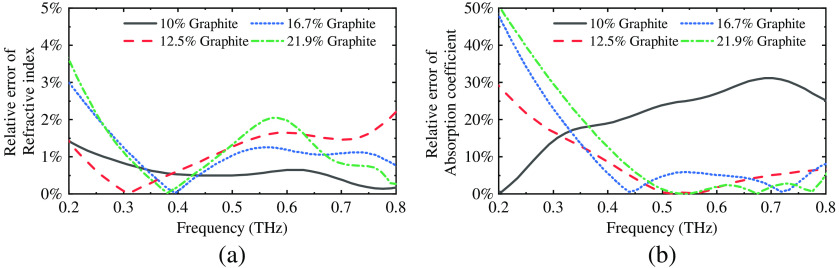
The relative error between measured and estimated optical properties [(a) refractive index and (b) absorption coefficient] of all phantoms. The overall performance of the BM for refractive index estimation is good with a relative error of up to 4%. For the absorption coefficient estimation, when the graphite concentration is higher than 10%, the BM is close to the experiment data in the frequency range of 0.4 to 0.8 THz with a relative error of up to 8%.

The BM is suitable for estimating the optical properties of a two-component solid mixture. The relative errors ($\delta =\frac{{\text{value}}_{\text{measured}}-{\text{value}}_{\text{estimation}}}{{\text{value}}_{\text{estimation}}}\times 100\%$) between measured and estimated optical properties are shown in Fig. 9. According to a prior paper,^15^ the requirement for the performance of a numerical model is that the relative error of estimation is lower than 10%. The overall performance of the BM for refractive index estimation is good with a relative error of up to 4%. For the absorption coefficient estimation of the phantoms with 12.5%, 16.7%, and 21.9%, the BM is close to the experiment data in the frequency range of 0.4 to 0.8 THz with a relative error of up to 8%. For the concentration of 10%, the BM’s estimation of absorption coefficient does not match the actual value very well, but the estimation of the refractive index is accurate. Additionally, for lower frequencies, the BM also has limited estimation accuracy because the absorption coefficient of graphite is very low at 0.2 THz. If the actual value of the y-intercept of the absorption curve is higher than 0, then we may have closer approaches.

As an initial research introducing a water-free phantom in the THz regime, for simplification purposes, we did not take the limitation of the BM into account and only focused on the reliability of using graphite as an absorber and the general similarity between phantom and target biotissue. However, we will investigate their effects in further studies that include measurements in the frequencies higher than 1 THz.

## 4 Conclusion

The results prove that the presented water-free phantom with 16.7% graphite can mimic the THz optical properties of cancerous oral tissue, and the phantom with 21.9% graphite is able to mimic healthy oral tissue in a limited frequency range of 0.6 to 0.8 THz. It is also revealed that the concentration of graphite significantly influences the optical properties of the phantom. The refractive index and the absorption coefficient increase along with the increase of graphite concentration. Additionally, the BM for spherical particles proved to be a useful model for numerical estimation of the optical properties of a phantom with certain graphite concentrations. These support the promise of developing phantoms for other tissues using the EMT. The proposed PVCP-graphite-based phantom also helps make further progress toward the application of THz technologies to oral cancer diagnosis and surgery in the future. Nonetheless, it is limited in the estimation of low frequency properties due to its dependence on the permittivity of each component. To achieve a phantom of other tissues, for example, breast cancer tissue, one or more additional materials are required since graphite with the concentration of 21.9% does not provide a high enough absorption coefficient in low frequencies. Theoretically, the permittivity of the additional materials should be able to be calculated by the BM if we consider the phantom with graphite as an entirety.
